# A Dynamic Transmission Model to Evaluate the Effectiveness of Infection Control Strategies

**DOI:** 10.1093/ofid/ofw247

**Published:** 2017-02-10

**Authors:** Karim Khader, Alun Thomas, W. Charles Huskins, Molly Leecaster, Yue Zhang, Tom Greene, Andrew Redd, Matthew H. Samore

**Affiliations:** 1 Informatics, Decision Enhancement, and Analytical Sciences 2.0 Center, VA Salt Lake City Health Care System, City, Utah; 2 Divisions of Epidemiology; 3 Genetic Epidemiology, University of Utah School of Medicine, Salt Lake City; 4 Division of Pediatric Infectious Diseases, Mayo Clinic, Rochester, Minnesota

**Keywords:** dynamic transmission model, infection control, randomized control trial

## Abstract

**Background:**

The advancement of knowledge about control of antibiotic resistance depends on the rigorous evaluation of alternative intervention strategies. The STAR*ICU trial examined the effects of active surveillance and expanded barrier precautions on acquisition of methicillin-resistant Staphylococcus aureus (MRSA) and vancomycin-resistant Enterococcus (VRE) in intensive care units. We report a reanalyses of the STAR*ICU trial using a Bayesian transmission modeling framework.

**Methods:**

The data included admission and discharge times and surveillance test times and results. Markov chain Monte Carlo stochastic integration was used to estimate the transmission rate, importation, false negativity, and clearance separately for MRSA and VRE. The primary outcome was the intervention effect, which when less than (or greater than) zero, indicated a decreased (or increased) transmission rate attributable to the intervention.

**Results:**

The transmission rate increased in both arms from pre- to postintervention (by 20% and 26% for MRSA and VRE). The estimated intervention effect was 0.00 (95% confidence interval [CI], −0.57 to 0.56) for MRSA and 0.05 (95% CI, −0.39 to 0.48) for VRE. Compared with MRSA, VRE had a higher transmission rate (preintervention, 0.0069 vs 0.0039; postintervention, 0.0087 vs 0.0046), higher importation probability (0.22 vs 0.17), and a lower clearance rate per colonized patient-day (0.016 vs 0.035).

**Conclusions:**

Transmission rates in the 2 treatment arms were statistically indistinguishable from the pre- to postintervention phase, consistent with the original analysis of the STAR*ICU trial. Our statistical framework was able to disentangle transmission from importation and account for imperfect testing. Epidemiological differences between VRE and MRSA were revealed.

Antibiotic-resistant pathogens are major causes of morbidity and mortality in healthcare settings such as acute care hospitals. A variety of strategies for control of resistant organisms have been proposed, including antibiotic stewardship, active surveillance, environmental decontamination, and treatment of colonization [1–8]. Yet, the pace of accumulation of evidence about the effectiveness of alternative types of interventions has been slow. Sources of bias and confounding that may explain variation in study results are incompletely understood.

Studies have typically entailed the comparison of rates of outcomes in groups of patients assigned to different interventions after either a quasiexperimental study design or cluster-randomized trial [9, 10]. The acquisition rate, defined as the number of admissions with a positive follow-up test after a negative baseline test per patient-day at risk, is the endpoint typically used in studies that are based on surveillance tests. A limitation of the acquisition rate is that it carries the assumption that tests are performed without error. Yet, there is abundant evidence that false-negative surveillance tests occur with appreciable frequency [11–16].

Another challenge in the analysis of infection outcomes is the need to deal with the dependence of events. Statistical methods that appropriately account for transmission are better able to distinguish the effects of different kinds of system changes on the spread of resistant organisms in human populations. Compared with the acquisition rate, the transmission rate parameter (transmission rate), defined as the rate of cross-infections per infectious individual is a more direct measure of the effectiveness of an intervention to improve source control via active surveillance. Unlike the acquisition rate, the transmission rate is not subjected to confounding because of importation of individuals who are infectious at the time of admission.

Statistical methods based on dynamic transmission models have been implemented previously [18–24]. Thus, estimating the transmission rate from data is not new in the statistical modeling of infectious diseases, but it is rare particularly in primary data analysis. Generally speaking, these statistical models are classified as either compartmental, which are used for aggregated data analysis, or patient-level models, which are used for analysis of data on individual patients. The choice between compartmental and patient-level models depends largely on the data available for analysis and the hypotheses being explored.

In this paper, we present the application of a dynamic transmission model to analyze the results of a cluster-randomized trial of active surveillance for control of MRSA and VRE [3]. The STAR*ICU trial evaluated the effect of active surveillance for MRSA and VRE colonization combined with the use of expanded barrier precautions (intervention) as compared with existing practice (control) on the incidence of MRSA or VRE colonization or infection in intensive care units (ICUs). Surveillance cultures were obtained from patients in all participating ICUs (10 intervention ICUs and 8 control ICUs); the results of the surveillance cultures were reported only to ICUs assigned to the intervention and during the intervention period. In intervention ICUs, patients who were colonized or infected with MRSA or VRE were assigned to care with contact precautions; all the other patients were assigned to care with universal gloving until their discharge or until surveillance cultures obtained at admission were reported to be negative. The primary endpoints in the STAR*ICU trial were MRSA and VRE acquisition rates. Postintervention acquisition rates, which were compared between treatment arms using baseline acquisition rates for adjustment, were not statistically different between the 2 arms.

Our rationale for reanalyzing the STAR*ICU trial was to generate deeper insights about variation in control of transmission across participating ICUs. The methods that we present here yielded an estimate of the transmission rate, embedded within a hierarchical modeling framework, extending recently published work [18]. Our approach accounts for the imperfect nature of surveillance tests and provides an estimate of the rate of clearance. Although dynamic transmission models have been implemented previously, to our knowledge, this is the first implementation of a hierarchical modeling framework used to examine the variation in transmission across multiple facilities, and it is the first effort at using dynamic transmission models for the purpose of estimating clearance rates.

## METHODS

### Data

We performed a retrospective analysis of data originally collected as part of the STAR*ICU trial. The data were collected during the period from April 2005 to August 2006 and included 20 945 patient-admissions admitted into one of the 18 participating ICUs. Nasal and perianal surveillance swabs were collected at the time of admission to the ICU, weekly thereafter, and on discharge from the ICU. Surveillance swabs were not collected for short-stay patients (ICU stay <3 days) in the original STAR*ICU trial, except for a random sample, which was used to estimate admission prevalence in the original study. However, swabs were collected from all long-stay patients (ICU stay ≥3 days), resulting in approximately 60% of all admissions to the ICU having at least 1 swab for MRSA and VRE. The observed data that we used in the transmission model were ICU identifier, ICU study-arm (control vs intervention), patient identifier, admission and discharge times, and surveillance culture times and results.

### Overview

The transmission model incorporated 2 main components, a within-ICU level component, which was nested in a between-ICU level component. The within-ICU component modeled patient movement into and out of the ICUs, colonization and clearance within the ICUs, and incorporated surveillance test data to inform the transmission dynamics. The between-ICU component was a model that specified the variation of model parameters across the ICUs.

A key feature of the between-ICU component of the transmission model was the stipulation of a hierarchical model for the pre- and postintervention transmission rates. Under the hierarchical model, the transmission rates were assumed to vary across the ICUs according to a common probability distribution in which the mean log-transformed postintervention transmission rate depended on the ICU’s assigned treatment. In particular, ICU-specific transmission rates were not modeled as fixed parameters, but they were modeled as log-normal random variables having a common mean and covariance with ICUs in the same study arm. The use of a hierarchical model for the transmission rates allowed us to characterize variation in transmission across ICUs and improved precision of ICU-specific transmission rate estimates via statistical shrinkage towards the overall mean log transmission rate across the ICUs. The hierarchical framework expressed statistical inferences for the treatment effect relative to the variation in transmission rates across the study ICUs. In this way, the Bayesian credible interval for the treatment effect on the transmission rate applied to the broader population of ICUs, and it extended inferences beyond the specific set of ICUs included in the study.

The models for MRSA and VRE were implemented independently within the Bayesian modeling framework, and parameter estimation was based on Markov chain Monte Carlo (MCMC) methods. In the next 2 sections, we give an overview of the transmission model and its assumptions. For additional details on the modeling assumptions and formulas, see the Supplementary Material.

### Within-Intensive Care Unit Level Model

**Figure 1.**
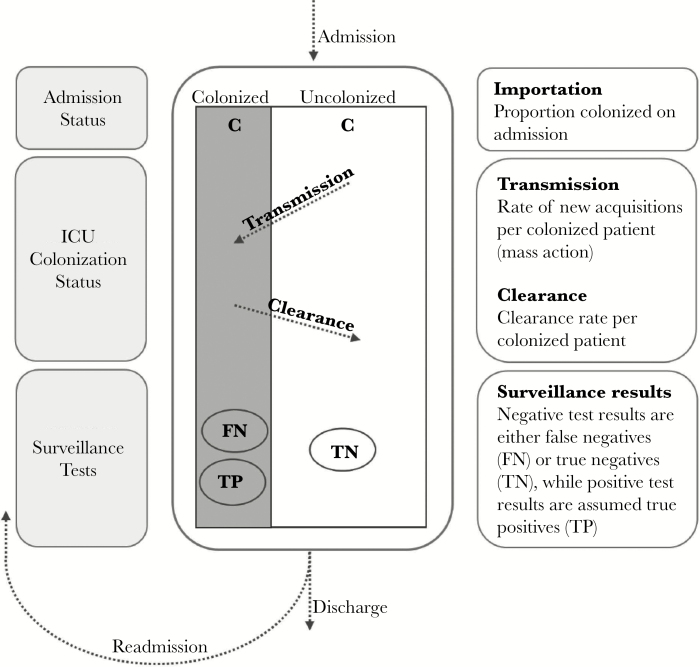
Diagram illustrating the underlying within-intensive care unit (ICU) transmission model that forms the basis for the full Bayesian transmission model incorporating the intervention across all ICUs.

A schematic illustrating the within-ICU model is provided in Figure 1. Upon admission into the ICU, patients were classified as either colonized (an importation) or uncolonized. Although patients were in the ICU, uncolonized patients became colonized according to the law of mass-action [ie, rate of new acquisitions per contact with a colonized patient = (transmission rate) × (no. of susceptible patients)], and colonized patients lost colonization at a constant “clearance rate”. The clearance rate represented generic loss of carriage, and it did not reflect a specific mechanism of clearance. Because decolonization was not part of the ICU intervention, but reflected ICU-specific practices, clearance rates were assumed to be ICU-specific. For a given patient stay, there were no constraints on the number of times that the patient could move between colonized and uncolonized states; however, the number of changes in colonization status was influenced by the patient’s length of stay, the model parameters, and the surveillance culture results. Although not technically necessary, we made the common simplifying assumption of no false positives. Therefore, the model interpreted all positive cultures as true positives, whereas negative cultures represented either true negatives or false negatives. At the time of discharge, patients were removed from the ICU and no longer contributed to the dynamics within the ICU. For patients readmitted to the ICU, their colonization status at their time of readmission was dependent on their prior discharge colonization status. A 2-state continuous-time Markov chain was used to model patient’s change in colonization status between consecutive ICU stays. The model provided an estimate of importation probability, defined here as the prevalence of colonization for first admissions or, equivalently, the limiting probability of colonization at the time of readmission, given a sufficiently long time after discharge.

### Between-Intensive Care Unit Level Model

Culture sensitivity parameters for MRSA and VRE were assumed to be the same across all 18 ICUs; part of the rationale for this simplifying assumption was that all surveillance cultures were sent to a common laboratory. All 18 ICUs were assumed to have a common mean transmission rate during the preintervention period and a potentially different mean transmission rate during the postintervention period, modeled independent of the intervention. The reason we included both pre- and postintervention mean transmission rates was to control for temporal trends in transmission rates across all ICUs. The estimated intervention effect parameter was modeled as an additive effect on the mean log-transmission rate in the intervention ICUs during the intervention period, thus the exponentiated intervention effect parameter can be interpreted as the relative change in the transmission rate. An estimated intervention effect parameter greater than zero denotes an increase in mean transmission rate, and an intervention effect parameter less than zero indicates a decrease in the transmission rate in the intervention ICUs during the intervention period compared with the control ICUs.

### Estimation

Estimation within each iteration of the MCMC consisted of generating a new sample of both the augmented data and the parameters, using either Gibbs sampling or Metropolis-Hastings sampling. Given the observed data and the current parameter values, new augmented data (or patient histories) consistent with the observed data and parameter values were proposed, and accepted, with probability that depends on the relative likelihood of the models with the proposed and current augmented data. If the proposed augmented data was not accepted, the current augmented data remained as the subsequent augmented data sample until the next iteration through the MCMC. Given the new augmented data, parameter values were proposed based on the observed data and the new augmented data. The process of updating the augmented data and parameter values was iterated, and resulted in a collection of parameter values, having a distribution consistent with the likelihood, conditioned on all observed and unobserved data. This collection of parameter values, known as the posterior distribution for the parameters, formed the basis for the point estimates and credible intervals. The implementation of this model was done in C++ and was run using an ASUS Ultrabook laptop. The posterior distributions were based on 50 000 samples with a burn-in of 10 000 samples, and these took approximately 2.7 hours to run. Additional details on the implementation of the MCMC estimation together with the code are included in the Supplementary Material.

### Analysis

We reported both posterior means and 95% credible intervals (CIs) for the parameters. The estimated intervention effect parameter with its CI served as the statistical test of the effect of assignment to the intervention arm in comparison to the control arm. In addition, we estimated the preintervention and postintervention mean transmission rates, examined variation in ICU-specific estimates of the transmission rate (pre- and postintervention), importation probability, and clearance rate, and estimated the surveillance culture false-negative probability. For importation and clearance, median and range of estimates across all ICUs were calculated.

## RESULTS

**Figure 2.**
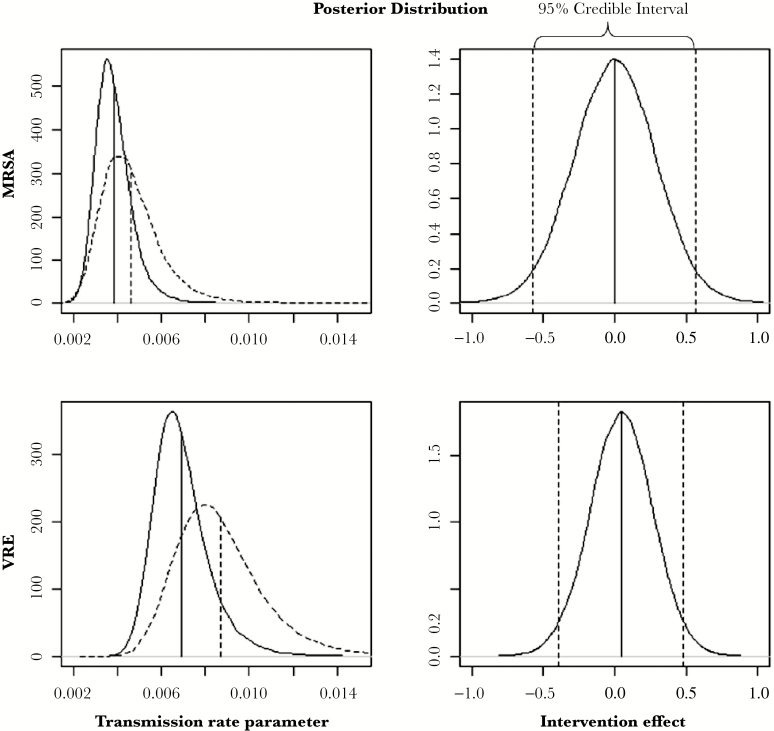
Posterior density for the pre- (solid curve) and postintervention (dashed curve) mean transmission rate parameter independent of the intervention (left), and the intervention effect parameter (right) for methicillin-resistant *Staphylococcus aureus* ([MRSA] top) and vancomycin-resistant *Enterococcus* ([VRE]; bottom).

The overall mean transmission rate (per infectious individual) rose from the pre- to the postintervention periods, from 0.0039 (95% CI, 0.0026–0.0058) to 0.0046 (95% CI, 0.0026–0.0079) for MRSA, representing a 20% increase. For VRE, mean transmission increased from 0.0069 (95% CI, 0.0049–0.0100) to 0.0087 (95% CI, 0.0056–0.0137), corresponding with a 26% rise (Figure 2). The estimated intervention effect parameter was 0.00 (95% CI, −0.57 to 0.56) for MRSA and 0.05 (95% CI, −0.39 to 0.48) (Figure 2) for VRE. There was a weak trend for a temporal association in mean transmission between the pre- and postintervention period, with an estimated correlation of 0.29 (95% CI, −0.26 to 0.72) for MRSA and 0.31 (95% CI, −0.19 to 0.71) for VRE.

**Figure 3.**
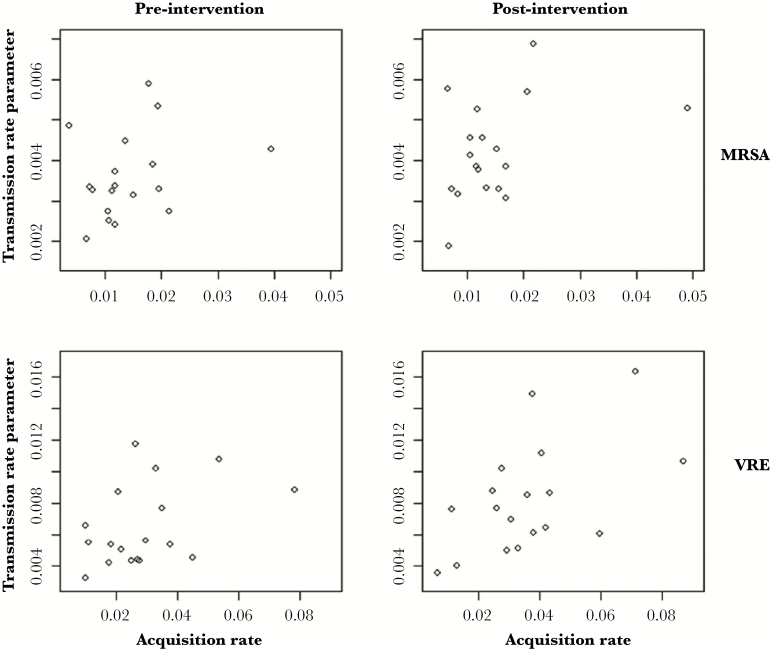
Relationship between the transmission rate parameter and the acquisition rate for methicillin-resistant *Staphylococcus aureus* ([MRSA] top) and vancomycin-resistant *Enterococcus* ([VRE]; bottom), during the preintervention period (left) and the postintervention period (right).

The ICU-specific estimates of the transmission rate demonstrated modest correlations with the acquisition rate estimates obtained in the analysis of the STAR*ICU trial (Figure 3). For MRSA, the correlation between the transmission rate and acquisition rate was estimated to be 0.29 (95% CI, −0.21 to 0.66) and 0.39 (95% CI, −0.09 to 0.73) during the pre- and postintervention periods, respectively, whereas for VRE, it was 0.43 (95% CI, −0.05 to 0.75) and 0.54 (95% CI, 0.10–0.80) during the pre- and postintervention periods, respectively. Thus, the correlations were only significantly different from zero for VRE during the postintervention period.

**Figure 4.**
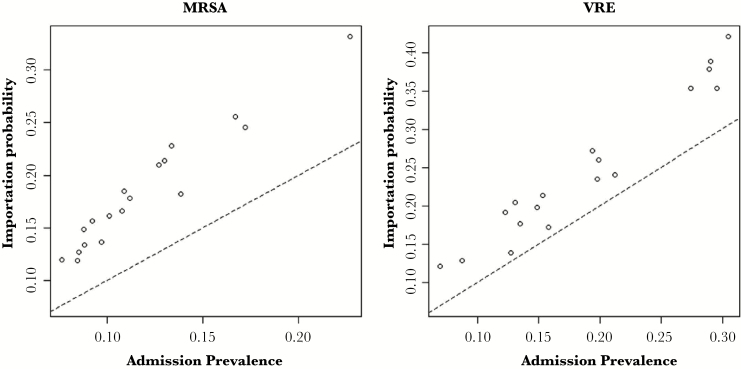
Relationship between admission prevalence (proportion of admission tests that are positive) and importation probability estimated by the transmission model for methicillin-resistant *Staphylococcus aureus* (MRSA) and vancomycin-resistant *Enterococcus* (VRE) by intensive care unit.

**Figure 5.**
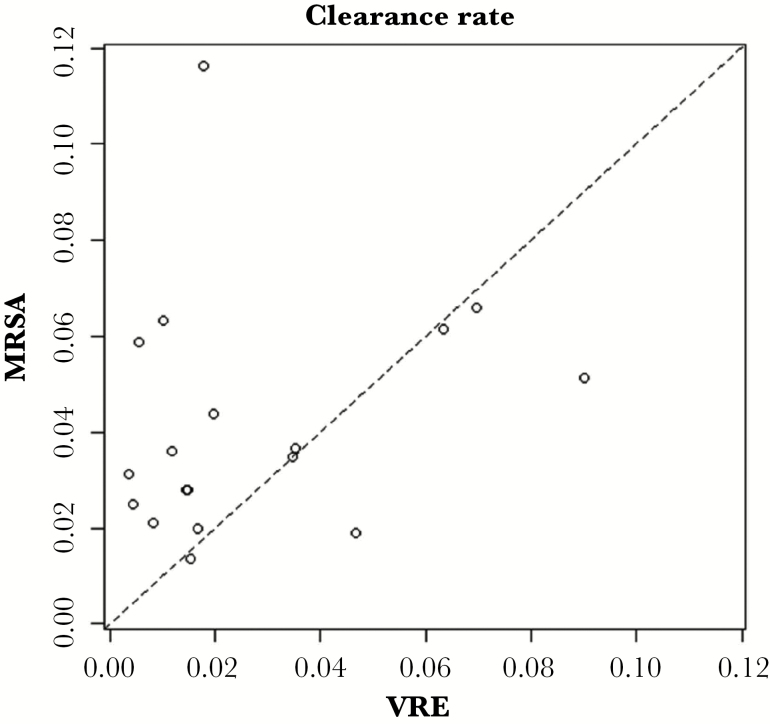
Relationship between clearance rate estimates for vancomycin-resistant *Enterococcus* (VRE) and methicillin-resistant *Staphylococcus aureus* (MRSA) by intensive care unit.

Our estimates of importation demonstrated wide variability across the 18 ICUs, similar to the variability in estimates for admission prevalence in the original study. The median estimate of MRSA importation was 0.17 (0.12–0.33), whereas the median estimate of VRE importation was 0.22 (0.12–0.42) (Figure 4). The median in-ICU clearance rate for MRSA was estimated to be 0.035 (0.013–0.116), corresponding with a median time to clearance of 20 days (6–53 days) (Figure 5). For VRE, the clearance rate across all ICUs was lower than that of MRSA, 0.016 (0.004–0.090), corresponding with a median time to clearance of 43 days (8–173 days). The estimated false-negative probability was 0.34 (95% CI, 0.31–0.38) for the MRSA surveillance culture and 0.31 (95% CI, 0.29–0.33) for the VRE surveillance culture.

## DISCUSSION

Our results support the conclusion of the primary analysis of the STAR*ICU trial [3], which illustrated that there was no evidence of an intervention effect. We found that transmission rates increased equivalently in both study arms from the pre- to postintervention period. The point estimates of the intervention effects were zero or close to zero for MRSA and VRE, respectively. In the original analysis, the adjusted combined acquisition rates demonstrated a minor, nonsignificant rise in the intervention arm compared with the control arm.

Our results demonstrated that the acquisition rate was a relatively poor predictor of the ICU-specific transmission rate. Stated differently, the acquisition rate varied substantially for any given estimate of the transmission rate. A number of factors likely contributed to this variability. The calculation of the acquisition rate is based on the count of the number of instances where an individual converts from a negative baseline test to a positive follow-up test. One of the problems with this determination is that it ignores situations where either the baseline test or follow-up test is falsely negative. Another limitation of the acquisition rate is that it fails to account for dependency between the number of infectious individuals present in a population and the force of infection, which is the rate of new infections (or colonizations) in susceptible members of the population.

By definition, the transmission rate offers a more precise assessment of the effect of an intervention to reduce cross-infection than more commonly used statistical measures. The distinctive dependence of infection events on the status of other members of the population renders conventional approaches to estimation of causal effects less valid [25]. The mass-action principle served as the theoretical foundation for the dynamic models used here and, in its general form, is supported by a wealth of empirical data. Moreover, estimation of the transmission rate from data establishes a direct link between statistical models and simulation models, which are often used to evaluate the consequences of alternative control policies through in silico experimentation.

Estimates of importation were consistently higher than those of admission prevalence (Figure 4), reflecting the reality that some negative admission surveillance cultures were likely false negatives. Consequently, estimates of admission prevalence based on the proportion of admission tests that are positive will often represent an underestimate of the underlying true burden of importation. The false-negative probability estimates for both MRSA and VRE surveillance cultures fell within the range observed in previous studies [11–16, 26, 27], as were the estimates of clearance rates [28–30]. Although clearance for both MRSA and VRE have been studied previously [28–34], prior studies have frequently defined clearance in terms of a fixed rule applied to surveillance cultures, which does not perfectly reflect the underlying truth, and hence are subject to misclassification. For example, a common definition for clearance has been 3 consecutive negative cultures over a fixed period of time. Such a definition ignores the fact that surveillance cultures are imperfect, and the level of colonization, which influences culture results, is a dynamic process changing over time. In contrast, our method treated clearance more realistically, as an unobserved and random process, which was imputed by the underlying dynamics of the transmission model, and thus required no assumption of a perfect correspondence between the underlying true colonization status and the observed data. Our study provides additional insights about differences observed between VRE and MRSA at the time of the original study that help to explain the observation of higher colonization prevalence for VRE than that of MRSA. We found that VRE tended to have a higher transmission rate, higher importation probability, and a lower clearance rate than MRSA. If the differences in transmission and clearance between VRE and MRSA represent facts that could have been generalized to the hospital or to the community, the higher importation probability of VRE compared with MRSA could have followed as a natural consequence.

The STAR*ICU trial was a cluster-randomized trial, so by design it did not need to explicitly account for patient-level or facility-level covariates. Consequently, our model excluded patient-level and ICU-level covariates to more directly contrast the results of our analysis with the results from the original study [3]. However, as has been done previously [19], patient-level and ICU-level covariates could be included in the model, which could potentially overcome a number of the obstacles that have been identified in previous work [5, 35–40]. Infection control interventions may have different effects across different ICUs, due to variation in intervention adherence, and patient mix. Controlling for variation in implementation in diverse ICU-settings may improve intervention effect estimates and provide a deeper understanding about which factors influence the performance of interventions.

In addition, this modeling framework can be used to explore, through virtual experiments, how the impact of interventions might vary under hypothetical intervention scenarios. Knowledge gained by such exploration could enhance the design of future intervention studies by providing an improved and more subtle understanding about what factors contribute to a successful intervention.

## CONCLUSIONS

We developed a dynamic transmission model that can be used for studying the impact of infection control interventions in settings where surveillance cultures are collected. The results of our analysis of the STAR*ICU trial data suggest no reduction of transmission for either MRSA or VRE due to the intervention, consistent with the original study. In addition, we found a broad range of estimates for importation and clearance rates, and estimates of the false-negative probability for surveillance cultures were consistent with other studies.

## Supplementary Data

Supplementary materials are available at *Open Forum Infectious Diseases* online. Consisting of data provided by the authors to benefit the reader, the posted materials are not copyedited and are the sole responsibility of the authors, so questions or comments should be addressed to the corresponding author.

## Supplementary Material

ofw247_suppl_khadermanuscriptofid_supplemental_material

ofw247_suppl_supplementaryfiles
