# Supplementary material for: A Dynamic Transmission Model to Evaluate the Effectiveness of Infection Control Strategies
Source: Open Forum Infect Dis. 2017 Feb 10;4(1):ofw247. doi: 10.1093/ofid/ofw247 (PMC5499871; doi:10.1093/ofid/ofw247)
Supplement: ofw247_suppl_khadermanuscriptofid_supplemental_material [file ofw247_suppl_khadermanuscriptofid_supplemental_material.pdf]

Supplemental Material

Transmission Model

The dynamic tranmission model that we implemented in this work is an extension of the model described previously in (Thomas et al. (2013)). The model extensions have been implemented in part to allow for a more general transmission model to account for multiple modeling assumptions, as well as to build a framework for specifically addressing the performance of the intervention in the STAR*ICU trial. The model was implemented within a Bayesian framework, depending on data D, which consists of observed data and is also augmented with unobserved data. Examples of the augmented data are the times of colonization or loss of colonization. We assume that there are N_I ICUs, and that the list of events ordered by time in ICU I is denoted by D_I ($D = \cup_I D_I$), and model parameters are represented by θ. We can express the likelihood for the augmented data D, given the model parameters θ as

$$\pi(D|\theta) = \prod_{I=1}^{N_I} \pi_I(D_I|\theta) = \prod_{I=1}^{N_I} \prod_{e \in D_I} g(e, t_e; \theta) h(t_e, t_{e^-}; \theta). \tag{1}$$

Here, we define t_e and t_e^- as the times of the events e and the prior event, e^-. $g(e, t_e; \theta)$ is a function that contributes to the likelihood according to the type of event (described in more detail below) e, while $h(t_e, t_{e^-}; \theta)$ represents probability that no event is observed between times t_{e^-} and t_e.

Model parameters and assumptions

The term $h(t_e, t_{e^-}; \theta)$ which represents the probability that no events occur between the consecutive events e^- and e, is given by the formula

$$h(t_e, t_{e^-}; \theta) = \exp\{S_I(t_{e^-}) C_I(t_{e^-}) \int_{t_{e^-}}^{t_e} \lambda_I(t) dt - C_I(t_{e^-}) \eta_I (t_e - t_{e^-})\}, \tag{2}$$

where $S_I(t)$ and $C_I(t)$ are the number of susceptible and colonized patients in ICU I at time t, $\lambda_I(t)$ is the transmission rate parameter in ICU I at time t, and η_I is the rate of loss of colonization for patients in ICU I.

Transmission

We assume that acquisitions in ICU I at time t for a given uncolonized individual occur at a rate $\lambda_I(t) C_I(t)$, which corresponds with the law of mass-action. In principal, we could allow $\lambda_I(t)$ to take on a number of functional forms, but in order to be consistent with the basic idea underlying the STAR*ICU trial, we assume that $\lambda_I(t)$ is piecewise constant taking one of two values,

$$\lambda_I(t) = \begin{cases} \lambda_I^{pre} & t \text{ is in the pre-intervention period} \\ \lambda_I^{post} & t \text{ is in the post-intervention period} \end{cases} \tag{3}$$

Consequently, the colonization rate for a given uncolonized individual is given by $g(e; \theta) = C_I(t_{e^-}) \lambda_I(t_e)$.

Clearance

We assume that for a given colonized patient in ICU I, they lose colonization at a constant rate η_I. Therefore, when a patient loses colonization in the model, the corresponding event contributes $g(e;\theta) = \eta_I$ to the likelihood.

Surveillance tests

Negative test results can correspond with either a true negative or a false negative, which depends on the unobserved colonization status reflected in the augmented data. We define $S(e)$ to be an indicator that the patient corresponding with event e is colonized at time t_e. Given this notation, the formula for the probability $g(e;\theta)$ of the negative test is given by $g(e;\theta) = \phi^{S(e)}(1-\phi)^{(1-S(e))}$, where ϕ is the probability of a false negative test.

Importation

The status of a patient at the time of admission contributes to the likelihood based on their probability of being colonized, or importing to the ICU at the time of admission. This importation probability depends on whether the admission event is the first admission or a readmission. Readmissions are modeled by a continuous time Markov chain in order to account for the possibility of switching colonization status during the period between consecutive admissions. In particular, between consecutive admissions, uncolonized become colonized at rate α_I and colonized lose colonization at rate γ_I. From these two parameters, we define the "steady-state", or first-admission importation probability as $\nu_I = \alpha_I/(\alpha_I + \gamma_I)$. Therefore, for a first admission event, we assume a constant importation probability ν_I into ICU I so that the contribution to the likelihood is

$$g(e;\theta) = \begin{cases} \nu_I, & S(e) = 1 \\ 1-\nu_I, & S(e) = 0 \end{cases} \tag{4}$$

For a patient whose admission event e is a readmission, their importation probability is computed using information from their previous discharge e_d. In particular, we use standard methods for computing transition probabilities of a homogeneous CTMC over the during the period of time from t_d to t_e. It is straighforward to compute the probability of importation at a readmission, and the contribution to the likelihood is given by

$$g(e;\theta) = \begin{cases} \nu_I[1-\exp\{-\Delta t \frac{\gamma_I}{1-\nu_I}\}]; & S(e) = 1,\, S(e_d) = 0 \\ \nu_I + (1-\nu_I)\exp\{-\Delta t \frac{\gamma_I}{1-\nu_I}\}; & S(e) = 1,\, S(e_d) = 1 \\ (1-\nu_I) + \nu_I \exp\{-\Delta t \frac{\gamma_I}{1-\nu_I}\}; & S(e) = 0,\, S(e_d) = 0 \\ (1-\nu_I)[1-\exp\{-\Delta t \frac{\gamma_I}{1-\nu_I}\}]; & S(e) = 0,\, S(e_d) = 1 \end{cases} \tag{5}$$

where $\Delta t = t_e - t_{e_d}$.

In-situ probability

At the time of the beginning of the study, there will be patients present in ICU I that were admitted prior to the beginning of the study. For those individuals, their probability of colonization at the beginning of the study is treated as a nuisance parameter, σ_I, called the *in-situ* probability. For a patient present at the onset of the study, their first observation which indicates presence in ICU I contributes $g(e;\theta) = \sigma_I^{S(e)}(1-\sigma_I)^{1-S(e)}$ to the likelihood.

Non-random events

For all other events e assumed to be observed without error, $g(e;\theta) = 1$.

Prior Distribution

Below we describe the prior distributions that help to complete the posterior distribution. We write the prior distribution as

$$\pi(\theta) = \pi(\phi) \cdot \prod_{I=1}^{N_I} \pi_I(\lambda_I^{pre}, \lambda_I^{post}, \mu^{pre}, \mu^{post}, \Sigma, \beta, \eta_I, \gamma_I, \alpha_I, \sigma_I) \tag{6}$$

$\pi_I(\cdot)$ can be further expressed as

$$\begin{aligned}\pi_I(\lambda_I^{pre}, \lambda_I^{post}, \mu^{pre}, \mu^{post}, \Sigma, \beta, \eta_I, \gamma_I, \alpha_I, \sigma_I) = \\ \pi(\lambda_I^{pre}, \lambda_I^{post}, |\mu^{pre}, \mu^{post}, \Sigma, \beta) \cdot \pi(\mu^{pre}, \mu^{post}, \Sigma, \beta) \cdot \pi(\eta_I) \cdot \pi(\gamma_I) \cdot \pi(\alpha_I) \cdot \pi(\sigma_I)\end{aligned} \tag{7}$$

Transmission parameters

We assume that $(\lambda_I^{pre}, \lambda_I^{pre})$ are bivariate log-normal random variables. In particular, we assume that

$$\log(\lambda_I^{pre}, \lambda_I^{post}) \sim \text{BVN}((\mu^{pre}, \mu^{post} + \chi(I) \cdot \beta), \Sigma).$$

Here, μ^{pre} and μ^{post} are the mean log-transmission rates during the pre-intervention and post-intervention periods respectively across all ICUs, both assumed independent of the intervention. We define $\chi(I)$ to be an indicator variable that ICU I is in the intervention arm of the study, β is the effect of the intervention on mean log-transmission, and Σ is the covariance matrix for $\{(\lambda_I^{pre}, \lambda_I^{post})\}_I$.

The mean log-transmission rates (μ^{pre}, μ^{pos}) and the corresponding covariance matrix (Σ) are assumed to have the normal-inverse-Wishart (NIW) distribution for their prior, which is the conjugate prior distribution corresponding with a multivariate normal likelihood.

The intervention effect parameter (β) is modeled having a normal prior distribution.

All other parameters

We use a gamma distribution as the prior distribution for outpatient colonization (α_I) and decolonization rates (γ_I), and for inpatient decolonization rates (η_I). The in-situ probability (σ_I) and false negative probability (ϕ) are given uninformative Beta priors.

MCMC Updates

Now we describe the sequence of steps used to update both the augmented data and the model parameters.

Augmented data

We use stochastic integration to account for the uncertainty in the augmented data set by updating the augmented data by sampling from the conditional distribution of each patient's history of colonization given the observed data, the current parameter values, and the current history of all other patients.

To sample the augmented data, we sequentially step through each patient admission and propose a new sequence of times that the patient might become colonized and decolonized. This proposal depends on the probability that the patient is an importation at the time of admission, as well as the current colonization and decolonization rates. The newly proposed augmented data is then accepted according to acceptance probability, following the Metropolis sampling scheme.

The complete history for a single patient admission can be specified by the initial state x (colonized or uncolonized), the number of times a change of state occurs y, and the sorted list of change times $u = (u_1, u_2, \ldots, u_y)$. Letting D^- represent the current state of the augmented data for all but the episode being updated, we can write the conditional posterior for this episode as $\pi(x, y, u|D^-, \theta)$ which can be calculated efficiently from the stored list of events as we describe them below. To implement an RJMCMC update, we first sample new values x' and y' from the distribution

$$\gamma^{x'}(1-\gamma)^{1-x'}\frac{\rho^{y'}e^{-\rho}}{y'!} \quad x' = 0, 1 \quad y' = 0, 1, 2, \ldots \tag{8}$$

so that x' and y' are independent Bernoulli and Poisson variates. Atypically for RJMCMC this proposal does not depend on the current state. The values of γ and ρ can be chosen arbitrarily to tune the mixing performance of the sampler. We then sample y' sorted $U(t_a, t_d)$ random variables $v_1, \ldots, v_{y'}$ where a and d are the admission and discharge events for the episode. That is, we sample $(v_1, \ldots, v_{y'})$ from the ordered uniform distribution

$$\frac{y'!}{(t_d - t_a)^{y'}} \quad t_a < v_1 < v_2 < \cdots < v_{y'} < t_d. \tag{9}$$

Thus, $(u_1, \ldots, u_y, v_1, \ldots, v_{y'})$ forms the complete set of continuous variables, active and auxiliary, associated with the current state. We transform this to the variables $(u'_1, \ldots, u_{y'}, v'_1, \ldots, v'_y)$ required for the new state by setting $u'_i = v_i, i = 1 \ldots y'$ and $v'_j = u_j, j = 1 \ldots y$, in effect simply switching the role of the active and auxiliary variables. This transformation is clearly reversible and has Jacobian 1. Hence, the proposed state x', y', u' is accepted with probability

$$\min\left\{1, \frac{\pi(x', y', u'|D^-, \theta)}{\pi(x, y, u|D^-, \theta)}\left(\frac{\gamma}{1-\gamma}\right)^{x-x'}\left(\frac{\rho}{t_d - t_a}\right)^{y-y'}\right\} \tag{10}$$

Transmission parameter updates

To update λ_I^{pre} and λ_I^{post} for each ICU I, we use Metropolis sampling. To improve mixing, we sequentially generate 10 Metropolis sample updates, keeping the 10th sample for the updated parameter values. For each Metropolis sample, we generate a pair of gamma$(2, 1)$ random variables. Each pair of gamma random variates are then accepted or rejected according to the standard Metropolis likelihood ratio. This process is repeated for each ICU I.

We sample $\mu = (\mu^{pre}, \mu^{post})$ and Σ using Gibbs samples by sampling directly from the normal inverse Wishart distribution. In particular, we make the following random draws:

$$\Sigma \sim \mathcal{W}^{-1}(\Psi, N_I)$$
$$\mu \sim \text{BVN}(\bar{\lambda}, \Sigma/N_I).$$

The parameters necessary for sampling from the above distribution are given by

$$\bar{\lambda} = \frac{1}{N_I} \sum_{I=1}^{N_I} \lambda'_I, \quad \text{where } \lambda'_I = (\lambda_I^{pre}, \lambda_I^{post} - \chi(I) \cdot \beta)$$

and

$$\Psi = \Psi_0 + S, \quad \text{where } S = \sum_{I=1}^{N_I} (\lambda'_I - \bar{\lambda})^T (\lambda'_I - \bar{\lambda}),$$

and we assume Ψ_0 to be the identity matrix.

We use Gibbs sampling to update β from a $\mathrm{N}(m_0, s_0^2)$ distribution. The parameters m_0 and s_0^2 are given by

$$m_0 = \frac{1}{N_{int}} \sum_{I \in \mathrm{Int}} (\lambda_I^{post} - \mu^{post})$$

and

$$s_0^2 = \frac{1}{N_{int}(N_{int} - 1)} \sum_{I \in \mathrm{Int}} \left[(\lambda_I^{post})^2 - (\mu^{post})^2 \right]$$

where N_{int} is the number of ICUs in the intervention arm of the study, and Int is the list of intervention ICUs.

Inpatient clearance update

To update the inpatient decolonization rate in ICU I, η_I, we use Gibbs sampling. We generate a Gamma(a_I, b_I) random variable for each I, where a_I gives the total number of decolonization events, and b_I gives the total patient time at risk for decolonization according to the augmented data. Updates for each ICU are made sequentially.

Outpatient parameter updates

To update the outpatient colonization rate α_I and decolonization rate γ_I, we use Metropolis sampling. For each of the two parameters, we propose a new parameter value by adding a standard normal random variable with small variance to the current parameter value. The proposed sample is accepted or rejected according to the standard Metropolis sampling scheme. To improve mixing, this process was iterated 10 times, keeping the 10th update for the new parameter value. Updates for each ICU are made sequentially.

False negative update

To update the value of the false negative parameter ϕ, we use Gibbs sampling, generating a beta(a, b) random variable where a is the number of false negative tests based on the augmented data, and b is the total number of tests in the dataset.

In-situ update

To update the in-situ probability of being colonized in ICU I σ_I, we use Gibbs sampling, generating a Beta(a_I, b_I) random variable for each I where a_I gives the total number of individuals present in ICU I at the beginning of the study who are colonized according to the augmented data, and b_I is the total number of individuals present in the ICU at the beginning of the study.

References

Thomas, Alun, Andrew Redd, Karim Khader, Molly Leecaster, Tom Greene, and Matthew Samore. 2013. "Efficient parameter estimation for models of healthcare-associated pathogen transmission in discrete and continuous time." *Mathematical Medicine and Biology : A Journal of the IMA*, 1–20. doi:10.1093/imammb/dqt021.
